# Management of walled-off pancreatic necrosis (WON) beyond the conventional step-up strategy: a retrospective cohort study

**DOI:** 10.1007/s00464-026-12607-w

**Published:** 2026-02-03

**Authors:** Mohamed Ebrahim, Morten Laksáfoss Lauritsen, David Fenger Schefte, Gitte Aabye Olsen, Srdan Novovic, John Gásdal Karstensen

**Affiliations:** 1 Pancreatitis Centre East, Gastro Unit, Copenhagen University Hospital Hvidovre, 2650 Hvidovre, Denmark; 2 Department of Clinical Medicine, University of Copenhagen, Copenhagen, Denmark

**Keywords:** Colonoscopy, Endosonography, Pancreatic fistula, Pancreatic necrosis, Pancreatitis, Acute necrotizing

## Abstract

**Objectives:**

Endoscopic transmural drainage and necrosectomy (ETDN) is a well-established technique for managing walled-off pancreatic necrosis (WON). However, ETDN may be insufficient in some cases, necessitating adjunctive techniques such as video-assisted retroperitoneal debridement (VARD) or endoscopic ultrasound-guided transcolonic drainage and necrosectomy (EUS-TC). The optimal step-up strategy in these patients remains unclear. We aimed to assess management of WON beyond the reach of ETDN.

**Methods:**

Patients with WON undergoing EUS-TC or VARD were identified from a prospectively maintained database between 2017 and 2024.

**Results:**

Forty-five consecutive patients (median age 53 years; median WON size 24 cm) were included, with a median follow-up of 19 months. Thirty-three patients (73%) underwent VARD, and 12 patients (27%) underwent EUS-TC. The median length of stay (LOS) was 85 days (IQR 48–126) for the VARD group and 74 days (IQR 40–152) for the EUS-TC group, and did not differ significantly (p = 0.818). In-hospital mortality was 18% (n = 8), all due to organ failure, with no difference between the two groups, p = 0.661. Thirty-seven (82%) patients achieved complete resolution (28 VARD, 9 EUS-TC). Overall, 10 (27%) patients (eight in VARD group and 3 in EUS-TC group, one patient was treated with both VARD and EUS-TC) developed 12 external fistulas (10 enterocutaneous (six colo-cutaneous, four small bowel) and two pancreatico-cutaneous. All pancreatico-cutaneous fistulas were managed conservatively, whereas all six patients with colo-cutaneous fistulas necessitated intervention.

**Conclusions:**

EUS-TC and VARD are both viable options for managing transgastrically inaccessible WON, although management remains associated with substantial LOS and external fistula formation.

**Graphical Abstract:**

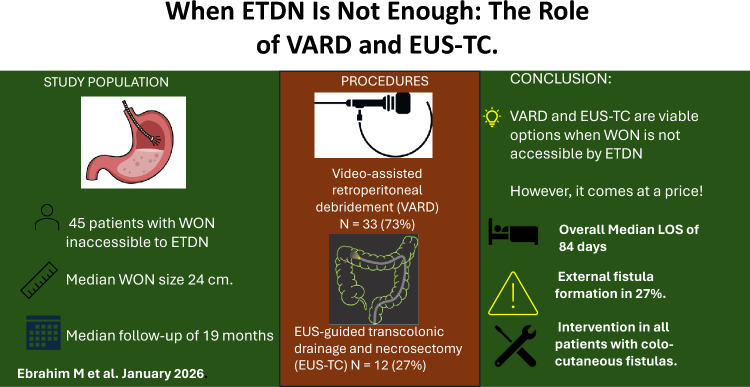

**Supplementary Information:**

The online version contains supplementary material available at 10.1007/s00464-026-12607-w.

Walled-off pancreatic necrosis (WON) is a late complication of acute necrotizing pancreatitis, associated with significant morbidity and mortality [1–3]. The step-up approach, with endoscopic transgastric drainage and necrosectomy (ETDN), has become a cornerstone for treating symptomatic WON, leading to improved clinical outcomes [4–8]. However, stepping up to auxiliary techniques such as video-assisted retroperitoneal debridement (VARD) or endoscopic ultrasound-guided transcolonic drainage and necrosectomy (EUS-TC) may be used in complex collections deemed inaccessible by the transgastric route—e.g., collections extending to the pelvic cavity, mesenteric root, or subphrenic spaces, or in a combined multi-gate approach as part of an accelerated treatment regime [9–13]. Despite an unmet need, algorithms to guide management of complex WON not amenable to conventional ETDN are scarce, and management remains subject to institutional variation [14–17]. While VARD is known to carry a considerable risk of percutaneous fistula formation, to date, no study has evaluated the risk of fistula formation in patients managed by EUS-TC [7, 8, 13].

Therefore, we aimed to evaluate the utility of VARD and EUS-TC as step-up strategies for the management of WON beyond the reach of conventional ETDN drainage, or in cases where ETDN was insufficient, in a multidisciplinary tertiary referral setting.

## Methods

### Patients and study design

This study was conducted as a retrospective observational cohort study at Pancreatitis Centre East (PACE), Copenhagen University Hospital Hvidovre, Denmark. Consecutive patients managed with EUS-TC or VARD were included in the following study period: January 1st, 2017 (when the first VARD was performed) to January 1st, 2024. Indications for treatment included infection, biliary obstruction, gastric outlet obstruction, and intractable pain. Patients with chronic pancreatitis, pregnancy, previous surgical or endoscopic drainage, or necrosectomy procedures were excluded. Clinical and radiological data, including treatment indications, were prospectively collected at the onset of acute pancreatitis, during treatment, and throughout follow-up in a dedicated database.

### Definitions

WON was defined according to the Revised Atlanta Classification as a fluid collection with a demarcated wall developing ≥ 4 weeks following acute necrotizing pancreatitis [18]. Infection in WON was suspected when I) Patient with WON presented with infection after other causes of infection had been ruled out. II) Presence of air in a WON collection on CECT (contrast-enhanced computed tomography). The technical feasibility of a procedure was defined as accomplishment of drainage and/or necrosectomy during the procedure. Clinical success was defined as regression of the WON at discharge, proven by CECT. Pancreatico-cutaneous fistula was defined as the presence of fluid at a percutaneous drain site or surgical wound with an amylase level exceeding the upper standard limit of serum amylase by three times or more, irrespective of drain volume [8]. A persistent pancreatico-cutaneous fistula was defined as a symptomatic fistula that did not resolve within six months following treatment of WON [7]. An enterocutaneous fistula was defined as output from a percutaneous drain site or surgical wound, either from the small or large bowel (colo-cutaneous fistula). CECT, fluoroscopy with fistulography, gastroscopy, or colonoscopy confirmed the diagnosis. All data regarding fistula outcomes were reviewed independently by four authors (ME, ML, DFS, SN).

### Diagnostic imaging before intervention

To offer a qualitative assessment of disease severity, the CT Severity Index (CTSI), the modified CT Severity Index (mCTSI) (calculated by use of CECT in the early phase of AP), and the QNI classification system (calculated at the time of index intervention) were included [19–21].

Patients were stratified as high-risk according to the QNI classification if they had necrosis involving ≥ three quadrants or two quadrants with ≥ 30% necrosis or one quadrant with ≥ 60% necrosis and documented infection [19]. CECT was performed in all patients before the index drainage procedure and discussed at a multidisciplinary pancreas team (MDT) meeting.

### MDT

Considering the tertiary referral structure of our center, MDT meetings are held weekly, starting before the patient's arrival, which enables a tailored approach to be established. These meetings are attended by a team composed of dedicated acute care surgeons, radiologists, microbiologists, advanced endoscopists, pancreatologists, and intensivists, who collaboratively determine the most appropriate management strategy for each patient. This approach ensures that both surgical and endoscopic options are considered.

### Drainage procedures

The EUS-guided transgastric and transcolonic drainage and necrosectomy approaches applied at our center have been thoroughly described in earlier publications, with clear documentation of indications for EUS-TC and/or VARD [12, 17, 22, 23]. All procedures were performed by the same team of acute care surgeons/advanced endoscopists during the study period.

### Follow-up

All patients were followed up in our outpatient clinic for a minimum of 1 year, during which surgical incisions were assessed, and CECT scans were evaluated.

### Primary and secondary outcomes

The primary outcomes were in-hospital mortality, length of stay (LOS), and procedure-related adverse events of VARD and EUS-TC.

Secondary outcomes included external fistula formation within one year after discharge and the need for surgical or endoscopic treatment of the fistula within one year of its formation.

### Statistical analysis

Continuous data are presented as medians with interquartile ranges (IQR), while categorical data are reported as counts and percentages. Group comparisons were conducted using Fisher’s exact test for categorical variables and the Mann–Whitney U test for continuous variables. All tests were two-tailed with a significance level of 5%. Statistical analyses were performed using R software version 4.2.2 (R Core Team 2022; Vienna, Austria) and the ‘gtsummary’ package [24, 25].

### Approvals

Permission for this study was granted by the Center for Regional Development, Capital Region of Denmark (ID no. R-20075169). No consent from the Ethics Committee was required as the study was purely registry-based. The study was reported according to The Strengthening the Reporting of Observational Studies in Epidemiology (STROBE) guidelines [26].

## Results

### Baseline demographics

**Fig. 1:**
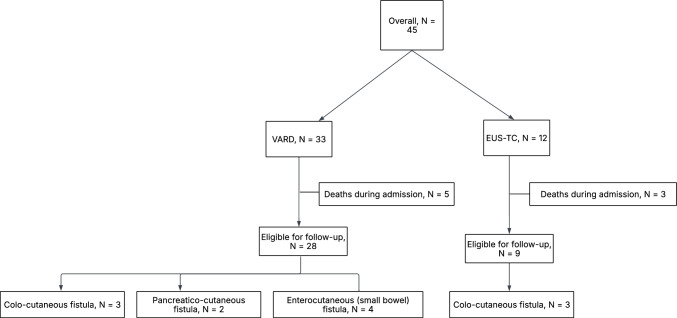
Flowchart showing management and fistula-related outcomes in patients with walled-off pancreatic necrosis (WON) managed with either video-assisted retroperitoneal debridement (VARD) or endoscopic ultrasound-guided transcolonic drainage and necrosectomy (EUS-TC). Overall, 10 unique patients (27%) developed fistulas. Notably, one patient (3%) underwent both VARD and EUS-TC and developed fistulas related to each procedure (pancreatico-cutaneous for VARD and colo-cutaneous for EUS-TC), counted in both groups

Of 251 patients undergoing intervention for WON during the study period, 45 (18%) were managed with EUS-TC or VARD (Fig. 1). Baseline data are shown in Table 1. Baseline characteristics of the two groups were similar, except for age, which was higher in the VARD group (57 vs. 44 years, p = 0.036).

**Table 1:** Baseline patient characteristics at time of onset of acute necrotizing pancreatitis

	Overall, N = 45	EUS-TC, N = 12	VARD, N = 33	p-value
Age, years, median (IQR)	53 (39–64)	44 (37–53)	57 (48–67)	0.036
Male, n (%)	24 (53%)	8 (67)	16 (49)	0.501
ASA-score, median (IQR)	2 (2–3)	2 (2–3)	2 (2–3)	0.224
*Etiology, n (%)*				0.428
Gallstone	14 (31)	6 (50)	8 (24)	
ERCP	19 (42)	3 (25)	16 (48)	
Alcohol	7 (16)	2 (17)	5 (15)	
Hyperlipidemia	2 (4)	0	2 (6)	
Idiopathic/other	3 (7)	1 (9)	2 (6)	
Days from onset to index intervention, median (IQR)	32 (22–45)	28 (26–40)	32 (22–46)	0.837
Need for ICU before index intervention, n (%)	30 (67)	8 (67)	22 (67)	1
WON size at index intervention, cm, median (IQR)	24 (20–29)	24 (21–27)	23 (20–30)	0.878
CTSI, median (IQR) ^*^	4 (3–8)	3.5 (3–5)	5 (3–8)	0.593
mCTSI, median (IQR) ^*^	6 (4–8)	5 (4–6.5)	6 (4–10)	0.175
QNI high-risk group^‡^	31 (69)	7 (58)	24 (73)	0.470
DPDS	8 (18)	5 (15)	3 (25)	0.661
Infected WON^‡^	43 (96)	12 (100)	31 (94)	0.939

*IQR* interquartile range, *ASA* American Society of Anesthesiology, *ERCP* endoscopic retrograde cholangiopancreatography, *ICU* Intensive Care Unit; *WON* walled-off pancreatic necrosis, *CTSI* CT severity index, *mCTSI* Modified CTSI, *QNI* Quadrant Necrosis Infection, *DPDS* Disconnected pancreatic duct syndrome

^*^CTSI and mCTSI were calculated from the first CT scan obtained during the early phase of acute pancreatitis, typically within two weeks

^‡^High-risk group was defined as involvement of ≥ 3 quadrants or two quadrants with ≥ 30% necrosis or one quadrant with ≥ 60% necrosis and documented infection. ^‡^ Culture-verified Infection

### EUS-TC and VARD procedures

Of patients being treated with either EUS-TC or VARD, the index intervention was ETDN in 27 (60%) patients, VARD in 12 (27%), and a combined multi-gate approach in six (13%) patients (four patients underwent combined ETDN and VARD, and two patients combined ETDN and EUS-TC) (Table 2). Four (9%) patients were managed solely with VARD. VARD was selected as the primary intervention in 12 (27%), as pre-interventional CECT imaging suggested that ETDN would be unfeasible due to the challenging anatomical location of the WON. EUS-TC was used as a supplementary intervention due to insufficient ETDN (n = 9) or VARD (n = 3). The indications for EUS-TC and VARD differed: VARD was used to accelerate treatment in 15 (45%) patients, compared with only 2 (17%) in the EUS-TC group (p = 0.096). In contrast, EUS-TC was used to access collections not reachable by index intervention in 10 (83%) patients, compared to 18 (54%) patients in the VARD group (p = 0.096) (Table 2).

**Table 2:** Procedural characteristics and outcomes

	Overall, N = 45	EUS-TC, N = 12	VARD, N = 33	p-value
*Indications for additional procedures*				
Collection not accessible via index intervention	28 (62)	10 (83)	18 (54)	0.096
Acceleration of treatment/multi-gate approach	17 (38)	2 (17)	15 (46)	0.096
*Index intervention*				
ETDN	27 (60)	10 (75)	17 (52)	0.085
VARD	12 (27)	0	12 (36)	0.02
Combined intervention	6 (13)	2 (17)	4 (12)	0.650
Total number of procedures, including additional procedures, median (IQR)	5 (4–6)	5.50 (4–6)	5 (3–9)	0.895
Need for open surgical necrosectomy	2 (4)	1 (8)	1 (3)	0.466
Technical success, n (%)	42 (93)	10 (83)	33 (100)	0.066
Clinical success, n (%)	39 (87)	11 (92)	28 (85)	0.83
LOS in days, median (IQR)	84 (46–126)	74 (40–152)	85 (48–126)	0.818
In-hospital mortality, n (%)	8 (18)	3 (25)	5 (15)	0.661
*Procedure-related adverse events, n (%)*				
Bowel perforation during intervention	2 (7)	2 (17)	0	0.07
Bleeding requiring intervention	1 (2)	0	1 (3)	1.00

*ETDN* Endoscopic transgastric drainage and necrosectomy, *VARD* Video-assisted retroperitoneal debridement, *EUS-TC* Endoscopic ultrasound-guided transcolonic drainage and necrosectomy; *IQR* Interquartile range, *LOS* Length of stay

### Clinical outcomes

The median LOS for the entire cohort was 84 days (IQR 46–126) with no significant difference between the two groups (p = 0.818) (Table 2). Overall, eight (18%) patients died during hospital admission, all due to multi-organ failure. Technical success was achieved in all but two (17%) patients who underwent EUS-TC. In one case, drainage was considered impossible due to interposing vessels, and the patient underwent VARD instead. Technical success was achieved in all patients who underwent VARD (Table 2).

### Procedure-related adverse events

Colonic perforation occurred due to stent misdeployment in one patient during EUS-TC, necessitating a laparotomy for bowel closure without stoma formation and an uneventful recovery (Supplementary Table 1). One patient in the VARD cohort had a bleeding complication (subcutaneous bleeding) requiring re-exploration (Table 2).

### Surgical intervention

Open surgical necrosectomy was performed in two (4%) patients due to continuous clinical deterioration with refractory sepsis despite minimally invasive approaches (Table 2). Surgery due to other causes is presented in Supplementary Table 1.

### Outcomes at one-year follow-up

A total of 37 patients (82%) were eligible for follow-up. The median follow-up period was 19 months (IQR 14–25), and it was similar in both groups.

During follow-up, six (16%) patients died, although none of these deaths were related to WON. Overall, 10 (27%) patients (eight in the VARD group and three in the EUS-TC group (one patient was treated with both VARD and EUS-TC)) developed 12 external fistulas: 10 enterocutaneous (six colo-cutaneous, four small bowel) and two pancreatico-cutaneous fistulas (Table 2). One patient developed a persistent pancreatico-colo-cutaneous fistula following VARD and EUS-TC (counted in both groups, Fig. 1, Supplementary Table 2; Patient 2). Both pancreatico-cutaneous fistulas were managed conservatively, with one persistent fistula after discharge. This fistula closed spontaneously within a year (Supplementary Table 2; Patient 2).

Colo-cutaneous fistulas occurred in three (33%) patients in the EUS-TC group versus three (11%) patients in the VARD group (p = 0.140). Of note, three (27%) patients initially managed with ETDN and VARD who later required EUS-TC to address collections not amenable to drainage developed fistulas. Importantly, all these fistulas preceded the EUS-TC intervention and were all related to VARD except for the single patient who underwent both VARD and EUS-TC and developed fistulas related to each procedure (Fig. 1). No enterocutaneous (small bowel) or pancreatico-cutaneous fistulas were observed in the group managed with EUS-TC, in contrast to VARD, where the former required surgery in three (75%) of four patients (Table 3).

**Table 3:** Fistula outcomes following treatment of walled-off necrosis with EUS-TC and VARD

	Overall, N = 37	EUS-TC, N = 9	VARD, N = 28	p-value
Colo-cutaneous fistula	6 (16)	3 (33)	3 (11)	0.140
Persistent colo-cutaneous fistula	4 (11)	2 (22)	2 (7)	0.243
Enterocutaneous fistula (small bowel)	4 (11)	0	4 (14)	0.553
Persistent enterocutaneous fistula (small bowel) ^*^	3 (8)	0	3 (11)	0.562
Pancreatico-cutaneous fistula	2 (5)	0	2 (7)	1
Persistent pancreatico-cutaneous fistula	1 (3)	0	1 (4)	1
Indications for surgery/endoscopy to achieve fistula closure within one-year following treatment, n (%)	9 (24)	3 (33)	6 (21)	0.657
Pancreatico-cutaneous fistula	0	0	0	
Colo-cutaneous fistula	6 (16)	3 (33)	3 (11)	
Enterocutaneous (small bowel) fistula	3 (8)	0	3 (11)	

*VARD* Video-assisted retroperitoneal debridement, *EUS-TC* Endoscopic ultrasound-guided transcolonic drainage and necrosectomy, *IQR* Interquartile range

^*^Two of the three patients with enterocutaneous (small bowel) fistulas developed the fistula following a laparotomy

## Discussion

This retrospective, single-center tertiary study showed that EUS-TC and VARD were technically feasible for managing complex WON collections beyond the reach of conventional ETDN and in cases where ETDN drainage and necrosectomy alone were insufficient.

In this study, we reported a median LOS of 84 days and an in-hospital mortality rate of 18%. Our median LOS exceeds previously reported estimates from our center, ranging from 32 to 53 days [1, 17, 23]. This difference is explained by the complexity of the included patients, characterized by extensive, multi-locular WON collections (median WON size of 24 cm, IQR 20–29) and the need for ICU management before the index intervention in 67% of cases. This further raises a question about the optimal management strategy for complex WON collections. To date, current guidelines have not endorsed a validated universal approach for complex WON not amenable to ETDN drainage [27–29]. Hence, management and the choice of step-up strategies for complex WON are subject to institutional factors, which further complicate decision-making [1, 15, 30]. The current step-up approach is labor-intensive with repetitive endoscopic drainage and only steps up to surgical approaches when the endoscopic strategy is deemed insufficient, potentially leading to prolonged hospitalization and increased morbidity [14, 16, 17, 30]. A recent randomized trial from our center showed a 53% reduction in LOS for patients with WON > 15 cm managed with an aggressive approach, including immediate necrosectomy at the index procedure, compared with the conventional step-up approach. In addition, none of the patients in the accelerated group required adjunctive VARD, compared with 25% of patients in the traditional step-up group [16]. We believe that a more aggressive approach with upfront endoscopic necrosectomy has a promising role in the management of complex WON, as direct necrosectomy may hinder compartmentalization of the WON into areas beyond the reach of ETDN, often requiring adjunctive step-up strategies such as percutaneous catheter drainage (PCD), VARD, or EUS-TC [16]. Whether the included patients would have benefited from an accelerated multi-modal drainage and necrosectomy approach at index endoscopy, and whether such a strategy could obviate the need for adjunctive techniques, is an open question that highlights the need for a well-powered multi-center randomized trial to address this subset of patients with WON in the future.

No patient in our group required surgery for the management of pancreatico-cutaneous fistulas, contrary to results from previous studies [4, 6–8]. These differences may be attributed to more frequent use of transgastric drainage in combination with percutaneous drainage techniques at our center. This approach enables internal diversion of pancreatic secretions in case of pancreatic duct leakage, which is common in patients with considerable intra-pancreatic necrosis [31]. Moreover, we routinely leave indwelling double-pigtail plastic stents for a year after resolution of WON, which likely reduces the risk of fistula formation in case of disconnected pancreatic duct syndrome (DPDS) [32]. While PCD and VARD are effective for managing necrotic collections not accessible by ETDN, they carry an inherent risk of external fistula formation and wound complications, which challenge management and are associated with substantial short and long-term morbidity [2, 4–8, 33].

In addition, current management of pancreatico-cutaneous fistulas following WON remains non-standardized, and most applied strategies lack formal validation [34–36]. Additionally, existing methods are mainly derived from pancreatic cancer surgery literature, where pancreatico-cutaneous fistulas have been studied extensively [37]. In the literature, various interventions have been explored, including placement of biodegradable stents in the main pancreatic duct, techniques for pancreatic stump closure (suture versus stapler), preoperative radiation to induce fibrosis of the pancreatic gland, pharmacological therapy, and restrictive use of prophylactic drains after pancreatic resections [38]. Nevertheless, these options do not apply to patients with acute necrotizing pancreatitis complicated by WON, as they typically present in a state of physiologic distress and catabolism due to ongoing organ failure [39]. Some groups practicing robotic management of WON support internal surgical drainage via Roux-en-Y fistulojejunostomy in cases with persistent pancreatico-cutaneous fistulas as a minimally invasive strategy [40]. However, long-term clinical follow-up data, including cost-effectiveness analyses of this strategy and head-to-head comparisons with an endoscopic approach, are currently lacking.

To our knowledge, the present study is the first to report data on the adjunctive use of EUS-TC and VARD in a subgroup of patients with WON in whom ETDN was insufficient. Moreover, it is the first study to report fistula outcomes for patients managed with EUS-TC. Certain limitations should be considered when interpreting the results. First, this was a retrospective single-center study conducted at a tertiary center. Second, during the study period, various endoscopic tools were introduced, including lumen-apposing metal stents and the EndoRotor system, while other modalities, such as placement of nasocystic drain, were gradually omitted. These advancements may have influenced the efficacy of endoscopic techniques. A significant strength of this study is its conduct at a tertiary center with extensive clinical experience in multi-modal management of WON, and structured clinical follow-up for at least one year after discharge, which ensures consistent assessment of long-term outcomes [1, 12, 17, 22, 23]. Additionally, we did not exclude patients with WON who developed complications due to acute necrotizing pancreatitis requiring surgery (e.g., abdominal compartment syndrome, bowel ischemia, and perforation) before the index VARD or EUS-TC to reflect the use of these techniques in a real-world clinical setting. Moreover, the presence of infection in 96% of our patients, combined with the large WON size and high QNI scores, highlights the severity of this subgroup.

This study suggests that both EUS-TC and VARD are viable treatment options in managing WON beyond the reach of conventional ETDN. EUS-TC appears to be a technically feasible adjunct tool in the step-up approach, completely averting the risk of pancreatico-cutaneous and enterocutaneous (small bowel) fistulas. However, in patients considered for EUS-TC, the increased risk of colo-cutaneous fistula warrants attention, particularly if VARD/PCD has been performed or is anticipated, or if a patient presents with a spontaneous fistula from WON to the skin.

In conclusion, this study illustrates the complexity of managing WON collections beyond the reach of ETDN. It highlights the importance of a versatile therapeutic armamentarium and the crucial role of a structured MDT in facilitating decision-making and optimizing outcomes for this subgroup of patients.

## Supplementary Information

Below is the link to the electronic supplementary material.

Supplementary file1 (DOCX 50 KB)

## Data Availability

Data supporting the findings of this study are available on reasonable request from the corresponding author.

## Abbreviations

CECT: Contrast-enhanced computed tomography
CTSI: CT severity index
DPDS: Disconnected pancreatic duct syndrome
ETDN: Endoscopic transgastric drainage and necrosectomy
ERCP: Endoscopic retrograde cholangiopancreatography
EUS-TC: Endoscopic ultrasound-guided transcolonic drainage and necrosectomy
ICU: Intensive care unit
LOS: Length of stay
mCTSI: Modified CT severity index
MDT: Multidisciplinary team
PCD: Percutaneous catheter drainage
QNI: Quadrant Necrosis Infection classification
VARD: Video-assisted retroperitoneal debridement
WON: Walled-off pancreatic necrosis
