# Supplementary material for: Management of walled-off pancreatic necrosis (WON) beyond the conventional step-up strategy: a retrospective cohort study
Source: Surg Endosc. 2026 Feb 3;40(4):3202–9. doi: 10.1007/s00464-026-12607-w (PMC13053370; doi:10.1007/s00464-026-12607-w)
Supplement: Supplementary file 1 — Supplementary file1 (DOCX 50 KB) [file 464_2026_12607_MOESM1_ESM.docx]

## Supplementary Table 1. Overview of Patients Undergoing Surgery for Disease-Related Complications or Adverse Events Following Minimally Invasive Management of WON.

| **Patient** | **Age (years) / Gender (M/F)** | **WON size in cm, QNI** | **DPDS** | **Index-intervention** | **Complication to the Index intervention** | **Indications for Surgery** | **LOS, days** | **Clinical outcome** |
| --- | --- | --- | --- | --- | --- | --- | --- | --- |
| 1 | 36, M | 40 cm, Q1,2,3,4N2I1 | Yes | Multi-gate ETDN + VARD | Perforation of the descending colon during subsequent ETDN with EndoRotor necrosectomy, initially managed with Endoclips. | Clinical deterioration with persistent organ failure despite minimally invasive intervention, leading to open necrosectomy.  During open necrosectomy, bleeding occurred, and post-operative ischemia of the right colon led to re-laparotomy with right colectomy and formation of an end-ileostomy.  Further complicated by a persistent enterocutaneous (small bowel) fistula. | 214 | Alive |
| 2 | 56, F | 24 cm, Q1,2,3,4N0I1 | No | Multigate ETDN+ EUS-TC | No | Ten days post-ERCP, the patient developed total colon ischemia. Subtotal colectomy with end-ileostomy was performed. Open surgical necrosectomy, multigate ETDN + EUS-TC, and repeated VARD procedures were performed. | 151 | Died of multi-organ failure |
| 3 | 55, F | 22 cm, Q2,4N0I1 | No | ETDN, EUS-TC as secondary intervention for inaccessible left paracolic WON | No | EUS-TC complicated by perforation of the descending colon, requiring explorative laparotomy with bowel suture; no stoma formed. | 30 | Alive |
| 4 | 36, M | 9 cm, Q2,4N1I1 | No | ETDN, EUS-TC as a secondary intervention for inaccessible WON. This patient developed abdominal compartment syndrome, and a decompressive laparotomy was performed. | Yes | Perforation of the colon at the splenic flexure during insertion of a double pigtail stent. Requiring a laparotomy with primary bowel closure. No stoma formed. | 39 | Alive |
| 5 | 62, F | 20 cm, Q1,2,3,4N0I1 | No | VARD and subsequent ETDN | No | Before admission to our unit and 16 days post-ERCP, the patient developed a small bowel perforation 100 cm distal to the ligament of Treitz due to stent migration. An end-jejunostomy was created. The patient underwent a laparotomy at our unit due to a distal blowout with cutaneous fistula formation. During the laparotomy, reversal of the end-jejunostomy and suturing of a small bowel perforation were performed. The patient developed anastomotic leakage, further requiring Percutaneous transhepatic biliary drainage (PTBD) and somatostatin analogue therapy. | 154 | Alive |
| 6 | 65, M | 15 cm, Q2N0I1 | No | ETDN followed by VARD | Yes | Bleeding following VARD necessitated re-VARD, where a minor subcutaneous bleeding was identified. However, the patient developed abdominal compartment syndrome, prompting a laparotomy that revealed diffuse retroperitoneal bleeding. Damage control surgery with packing was performed. | 23 | Died in the ICU due to septic shock with disseminated intravascular coagulation |
| 7 | 63, F | 23 cm, Q1,3N0I1 | No | VARD followed by ETDN and EUS-TC | No | Post-ERCP pancreatitis and duodenal perforation during stent removal were managed with laparotomy and duodenal repair. | 74 | This patient died under palliative care 196 days after diagnosis due to recurrence of cholangiocarcinoma. |
| 8 | 36, M | 32 cm, Q2,4N0I1 | No | ETDN with subsequent VARD | No | Post-ERCP pancreatitis after biopsy of a malignant biliary stenosis. Subsequent bleeding from the gastroduodenal artery was initially managed with angioembolization. Ongoing bleeding led to laparotomy, revealing a source 35 cm proximal to the J-pouch, requiring bowel resection and stoma creation. | 101 | The patient died under palliative care 360 days after discharge. |
| 9 | 55, M | 23 cm, Q1,2,3,4N0I0 | No | ETDN with subsequent bilateral VARD | Yes | Spontaneous non-persistent enterocutaneous fistula (small bowel, 70 cm distal to the ligament of Treitz).  Enterocutaneous non-persistent fistula from the ascending colon following VARD.  The patient underwent laparotomy with closure of the small bowel fistula and creation of a protective loop ileostomy. Due to continuous production from the colo-cutaneous fistula to the right VARD incision, laparotomy with closure of the fistula and reversal of the loop ileostomy was performed with no further complications. | 110 | Alive |

ERCP: endoscopic retrograde cholangiopancreatography; ICU: intensive care unit; WON: walled-off pancreatic necrosis; CTSI: CT severity index; mCTSI: modified CT severity index; QNI: quadrant (Q)—abdominal distribution, necrosis (N)—percentage necrosis in WON, infection (I)—positive blood culture and/or systemic inflammatory response syndrome (SIRS) with positive WON culture; ETDN: endoscopic transgastric drainage and necrosectomy; VARD: video-assisted retroperitoneal debridement; EUS-TC: endoscopic ultrasound-guided transcolonic drainage; PTBD: percutaneous transhepatic biliary drainage; LOS: length of stay; M/F: male/female; J-pouch: ileal pouch-anal anastomosis.

## Supplementary Table 2: Overview of Patients With Symptomatic External Fistulas Following Minimally Invasive Management of WON.

| **Patient** | **Age (years) / Gender (M/F)** | **WON size in cm, QNI** | **Disconnected pancreatic duct syndrome (DPDS)** | **Index-intervention** | **Complications** | **Fistula type, persistent fistula (yes, no)** | **Management of fistula** | **LOS** | **Clinical outcome** |
| --- | --- | --- | --- | --- | --- | --- | --- | --- | --- |
| 1 | 36, M | 40 cm, Q1,2,3,4N2I1 | Yes | Multi-gate ETDN + VARD | During subsequent ETDN drainage with EndoRotator, a perforation of the descending colon occurred and was closed endoscopically with Endoclips; however, during open surgical necrosectomy, the perforation was noticed and successfully sutured.  Postoperative ischemia of the right colon leading to re-laparotomy with right colectomy and formation of an end-ileostomy. | Enterocutaneous fistula (small bowel to midline) (yes) | Primary suture repair of the fistula and histoacryl injection were attempted without success. Fistula closure was finally achieved with a conservative strategy consisting of nil per os, somatostatin analogue therapy, percutaneous drainage, and PTBD. | 214 | Alive |
| 2 | 55, F | 22 cm, Q1,2,3,4N2I1 | Yes | ETDN, VARD, and EUS-TC, and laparotomy as a secondary intervention for inaccessible left paracolic WON. | During laparotomy, the patient underwent suture closure of an enterocutaneous (colo-cutaneous fistula) | A complex fistula system consisting of a pancreatico-colo-cutaneous fistula system (yes) | The colo-cutaneous fistula was sutured during laparotomy, followed by ERCP, aiming to place a stent in the main pancreatic duct to reduce output from the pancreatico-cutaneous fistula. ERCP revealed a disrupted pancreatic duct. Distal pancreatectomy with splenectomy and left hemicolectomy was considered; however, both fistulas resolved by conservative measures within a year. | 119 | Alive |
| 3 | 43, M | 29 cm, Q2,4N0I1 | No | ETDN with subsequent PCD and EUS-TC | Spontaneous enterocutaneous fistula (colo-cutaneous) arising from a left paracolic WON collection extending through the retroperitoneum, ultimately perforating the scrotal skin. | Enterocutaneous fistula (colo-cutaneous) (yes) | Despite the application of an OVESCO® over-the-scope-clip (OSTC®) and a laparoscopic diverting loop-ileostomy, the fistula persisted, necessitating a laparotomy with  Ablation of the fistulous tract using Gold Probe^TM^ Bipolar Haemostasis Device followed by successful suture closure of the fistula. | 100 | Alive |
| 4 | 53, M | 20 cm, Q1,2,4N1I1 | Yes | ETDN with subsequent PCD and EUS-TC | No | Enterocutaneous (colo-cutaneous) fistula development following PCD and EUS-TC (no) | Managed successfully by OVESCO clip | 97 | Alive |
| 5 | 28, F | 42 cm, Q1,2,3,4N0I1 | No | ETDN with subsequent VARD and EUS-TC |  | Complex fistula system consisting of a gastro-colo-cutaneous fistula following VARD (yes) | Managed successfully by laparotomy with ablation of the colo-cutaneous fistula tract using Gold Probe^TM^ Bipolar Haemostasis Device, followed by suture closure of the fistula. The gastric component of the fistula closed spontaneously after closure of the colo-cutaneous fistula. | 140 | Alive |
| 6 | 62, F | 20 cm, Q1,2,3,4N0I. | No | Bilateral VARD and subsequent endoscopic transduodenal necrosectomy for a paraduodenal extension of WON to the right paracolic gutter. |  | Enterocutaneous fistulas (small bowel).  Before admission to our unit and 16 days post-ERCP, the patient developed a small bowel perforation 100 cm distal to the ligament of Treitz due to stent migration. An end-jejunostomy was created. A distal blowout with enterocutaneous (small bowel) fistula formation (no) necessitated further laparotomy with reversal of the end-jejunostomy; during this laparotomy, a small bowel perforation was also sutured. The patient developed anastomotic leak, which was managed by PTBD and somatostatin analogue therapy.  In addition, the patient developed an enterocutaneous fistula to the VARD incision site, most probably an enterocutaneous fistula with connection to the transduodenal cavity (yes). | The non-persistent enterocutaneous (small bowel) fistula was managed by reversal of the end jejunostomy. However, the patient developed anastomotic leak, and conservative management with PTBD and somatostatin analogue was successful in achieving closure.  The persistent enterocutaneous fistula to the right VARD incision closed spontaneously within a year after removal of the percutaneous drain. | 154 | Alive |
| 7 | 67, F | 26 cm, Q1,2,4N1I1 | Yes | ETDN and subsequent VARD |  | Colonic stenosis, with enterocutaneous (colo-cutaneous) fistula to the VARD incision site. (no) | Fistula resolved upon insertion of a self-expanding metal stent through the colonic stenosis (SEMS) | 127 | Alive |
| 8 | 67, F | 23 cm, Q1,2,3N0I1 | No | VARD and subsequent ETDN |  | Pancreatico-cutaneous fistula to the VARD incision site (no) | Spontaneous resolution of the fistula at clinical follow-up 90 days following discharge. | 123 | Died of mesenteric ischemia two years after discharge. |
| 9 | 55, M | 23 cm, Q1,2,3,4N0I0 | No | ETDN and subsequent VARD | Spontaneous Enterocutaneous (small bowel fistula 70 cm distal to the ligament of Treitz) and an enterocutaneous (colo-cutaneous fistula from the ascending colon) following VARD | Enterocutaneous (colo-cutaneous) fistula from the ascending colon to the VARD incision site at the right side (yes)  Enterocutaneous fistula from the left VARD incision site to the small bowel (no) | The patient underwent laparotomy with closure of the small bowel fistula and creation of a protective loop ileostomy. Later, a planned laparotomy was performed due to continuous production from the right VARD incision (colo-cutaneous fistula). During this laparotomy, closure of the colonic fistula and reversal of the loop ileostomy were performed successfully. | 110 | Alive |
| 10 | 63, F | 31 cm, Q1,3N0I1 | No | VARD followed by ETDN and EUS-TC | Enterocutaneous fistula following duodenal perforation during initial ERCP | Enterocutaneous fistula between right VARD incision and duodenal perforation (yes) | Due to the patient’s poor condition, no therapy was offered to control the fistula. | 74 | This patient died 196 days after discharge due to recurrence of cholangiocarcinoma. |

M/F: male/female; WON: walled-off pancreatic necrosis; QNI: quadrant (Q)—abdominal distribution, necrosis (N)—percentage necrosis in WON, infection (I)—positive blood culture and/or systemic inflammatory response syndrome (SIRS) with positive WON culture; DPDS: disconnected pancreatic duct syndrome; ETDN: endoscopic transgastric drainage and necrosectomy; VARD: Video-assisted retroperitoneal debridement; EUS-TC: endoscopic ultrasound-guided transcolonic drainage; ERCP: endoscopic retrograde cholangiopancreatography; PTBD: Percutaneous transhepatic biliary drainage; OSTC®: over-the-scope clip; SEMS: self-expanding metal stent; LOS: length of stay.
